# Short-Term Synaptic Plasticity at Interneuronal Synapses Could Sculpt Rhythmic Motor Patterns

**DOI:** 10.3389/fncir.2016.00004

**Published:** 2016-02-03

**Authors:** Yan Jia, David Parker

**Affiliations:** Department of Physiology, Development and Neuroscience, University of CambridgeCambridge, UK

**Keywords:** spinal cord, synaptic plasticity, neuronal network, rhythmic, synaptic depression

## Abstract

The output of a neuronal network depends on the organization and functional properties of its component cells and synapses. While the characterization of synaptic properties has lagged cellular analyses, a potentially important aspect in rhythmically active networks is how network synapses affect, and are in turn affected by, network activity. This could lead to a potential circular interaction where short-term activity-dependent synaptic plasticity is both influenced by and influences the network output. The analysis of synaptic plasticity in the lamprey locomotor network was extended here to characterize the short-term plasticity of connections between network interneurons and to try and address its potential network role. Paired recordings from identified interneurons in quiescent networks showed synapse-specific synaptic properties and plasticity that supported the presence of two hemisegmental groups that could influence bursting: depression in an excitatory interneuron group, and facilitation in an inhibitory feedback circuit. The influence of activity-dependent synaptic plasticity on network activity was investigated experimentally by changing Ringer Ca^2+^ levels, and in a simple computer model. A potential caveat of the experimental analyses was that changes in Ringer Ca^2+^ (and compensatory adjustments in Mg^2+^ in some cases) could alter several other cellular and synaptic properties. Several of these properties were tested, and while there was some variability, these were not usually significantly affected by the Ringer changes. The experimental analyses suggested that depression of excitatory inputs had the strongest influence on the patterning of network activity. The simulation supported a role for this effect, and also suggested that the inhibitory facilitating group could modulate the influence of the excitatory synaptic depression. Short-term activity-dependent synaptic plasticity has not generally been considered in spinal cord models. These results provide further evidence for short-term plasticity between locomotor network interneurons. As this plasticity could influence the patterning of the network output it should be considered as a potential functional component of spinal cord networks.

## Introduction

Although the short-term activity-dependent plasticity of individual synapses has been studied extensively (Zucker and Regehr, 2002), its role within neuronal networks remains difficult to address (see O'Donovan and Rinzel, 1997; Nadim and Manor, 2000; Thomson, 2000a,b; Martinez et al., 2014). Specific forms of activity-dependent synaptic plasticity could in principle be evoked by different network outputs. In rhythmically active networks this could lead to a potential circular interaction where network activity could evoke synaptic plasticity that influences network activity. The analysis of this interaction is complicated by several factors. Firstly, effects must be characterized by making paired recordings from identified network neurons; secondly, effects should be examined in active networks, but single connections cannot typically be examined on a background of network activity (e.g., Nadim et al., 1999); and finally, linking synaptic effects to network outputs requires targeted modifications of specific synaptic properties, but the tools needed for this are currently lacking.

The lamprey locomotor network is a model system for network analyses (e.g., Grillner et al., 1995). The locomotor network model consists of inhibitory and excitatory interneurons in two identical hemisegmental networks. In the original formulation of the locomotor network scheme (see for example Grillner et al., 1995), each hemisegment has excitatory interneurons (EIN) that connect to motor neurons, the large crossed caudal (CC) inhibitory interneurons, and the large inhibitory lateral interneurons (LIN; Buchanan et al., 1989); the CC interneurons inhibit contralateral motor neurons, LINs, and other CC interneurons (Buchanan, 1982), as well as the EINs; and the LINs inhibit the CC interneurons and motor neurons (Rovainen, 1974; Buchanan, 1982). This network, and its modification (Grillner, 2003) after problems with the classical network scheme were highlighted (Parker, 2000), are claimed by Grillner to offer a fully characterized network organization. However, these claims, which are frequently cited in the field, are not supported by the literature. Rovainen (1983) highlighted problems with any proposed segmental role of the CC interneurons and LINs. Buchanan (1999) also raised concerns over the segmental role of the CC interneurons. Finally, the crossing inhibitory connections from the CC interneurons to the EINs lacks the claimed experimental support (Parker, 2006). In discussing issues with the CC and LINs, Rovainen (1983) and Buchanan (1999) highlighted the potential segmental role of their smaller homologs (the SiINs Buchanan and Grillner, 1988 and ScINs Ohta et al., 1991, respectively), with the CC and LINs possibly being intersegmental interneurons. Although the SiINs and ScINs have been examined, the connectivity of these cells, their functional properties, and their role in the network still remains poorly understood (Parker, 2006, 2010).

While interneuron inputs to motor neurons have been studied in some detail in the lamprey locomotor network, connections between network interneurons are generally poorly characterized. This reflects the difficulty of making paired recordings from the small interneurons that probably form the segmental locomotor network (see Parker, 2006). Paired recordings from premotor interneurons are rare in most spinal networks (the tadpole is an exception; Li et al., 2009), but this data is needed to understand network activity. While not considered in lamprey locomotor network models (e.g., Kozlov et al., 2001; but see Kozlov et al., 2009), several types of identified network synapses exhibit short-term activity-dependent plasticity in response to physiologically-relevant stimulation (Parker and Grillner, 1999, 2000; Parker, 2003a,b reviewed in Silberberg et al., 2005; Parker, 2007). EIN inputs to motor neurons have been studied in most detail. This connection typically depresses, but the properties of single EPSPs and their activity-dependent plasticity vary markedly, suggesting the presence of different functional sub-groups within this interneuron pool (Parker, 2003a, 2015). Connections between EINs have been identified (Parker and Grillner, 2000), but while these connections are probably essential in driving network activity, the small sample size (*n* = 4) is too small to allow characterization of the connection (connections between excitatory interneurons have been studied in detail in the tadpole spinal cord; e.g., Li et al., 2009). Synaptic plasticity has also only been examined in quiescent networks, and thus we do not know how it influences the network output.

Attempts have been made to address these gaps in this study. Firstly, the analysis of short-term synaptic plasticity at monosynaptic connections between network interneurons, including between EINs, has been extended to better characterize the connections; and secondly, the influence of activity-dependent synaptic plasticity on network activity has been examined experimentally and in a simple network model to examine how it could influence the network output. The results support the presence of functional motifs produced by the activity-dependent properties of network interneuron synapses, and also support a role for short-term plasticity as a functional component underlying the patterning of locomotor network activity. A preliminary account of the experimental analysis of altering depression on fictive network activity and the modeled effects has been reported previously (Jia and Parker, 2009).

## Materials and methods

Adult lampreys kept at 6°C were used. All maintenance and experimental procedures were approved by the local animal care committee and were performed under licence from the UK Home Office [Animals (Scientific Procedures) Act 1986]. For dissection, animals were anesthetized in MS222 and the spinal cord and notochord were removed from the trunk region of the body in oxygenated Ringer containing (in mM): 138 NaCl, 2.1 KCl, 1.8 CaCl_2_, 1.2 MgCl_2_, 4 glucose, 2 HEPES, 0.5 L-glutamine. The pH of the Ringer and of drug solutions was adjusted to 7.4 with 1 M NaOH. In corrected low Ca^2+^ Ringer, CaCl_2_, was reduced to 0.9 mM and in corrected high Ca^2+^ Ringer CaCl_2_ was increased to 3.6 mM, in both cases MgCl_2_was adjusted accordingly. In uncorrected Ringer the same changes in Ca^2+^ were made without adjusting Mg^2+^.

Synaptic properties were examined in pieces of spinal cord (~20 segments long) isolated from the notochord and placed in a Sylgard lined chamber superfused with Ringer at 10–12°C. Intracellular recordings were made using thin walled micropipettes filled with 3 M potassium acetate and 0.1 M potassium chloride. Motor neurons were identified by recording orthodromic spikes in ventral roots following current injection into their somata. Interneurons were identified by their ability to elicit monosynaptic EPSPs or IPSPs, respectively, in ipsilateral or contralateral postsynaptic cells (see Parker, 2003b). Connections between hemisegmental interneurons were found by recording from an excitatory (EIN) or inhibitory (SiIN) interneuron (Interneuron_1_) identified by its monosynaptic input to a postsynaptic neuron. The postsynaptic electrode was then withdrawn and used to find an interneuron that synapsed onto Interneuron_1_. Presynaptic interneurons were located 1–2 segments rostral to postsynaptic neurons. Monosynaptic connections were identified by their reliability and constant latency following presynaptic stimulation at 20 Hz (Berry and Pentreath, 1976). Glass suction electrodes with tips that covered the entire left or right half of the spinal cord were placed bilaterally on the spinal cord approximately 10 segments caudal to the cell recording site. The absence of orthodromic spikes in these electrodes when action potentials were evoked in the interneurons suggested that smaller interneurons rather than larger crossed caudal (CC) or lateral interneurons (LIN) were recorded (see Parker, 2003b). An Axoclamp 2A amplifier (Axon Instruments, California) was used for voltage recording and current injection. Data were acquired, stored, and analyzed on computer using an analog-to-digital interface (Digidata 1200, Axon Instruments, California) and Axon Instruments software (pClamp 9). The membrane potential was kept constant by injecting depolarizing or hyperpolarizing current using single electrode discontinuous current clamp (DCC; switching rate between ~2 KHz).

Activity-dependent plasticity was examined by stimulating the presynaptic interneuron using a train of five spikes evoked at 5, 10, and 20 Hz (this is physiologically-relevant stimulation; Buchanan and Cohen, 1982; Buchanan and Kasicki, 1995). Five trains were evoked at 20 s intervals at each frequency. The initial PSPs in the trains were used to measure low frequency-evoked inputs. PSP amplitudes were measured at the peak amplitude above or below the baseline immediately preceding the PSP (Parker, 2003a). Where possible, PSP rise times (10–90%) and half-widths were measured in Clampfit. Activity-dependent plasticity was assessed as PSP_5_/PSP_1_: depression was defined as PSP_5_ falling to at least 90% of PSP_1_, and facilitation as PSP_5_ being 110% or more of PSP_1_. Connections that did not match these criteria were classified as unchanged.

Fictive network activity was examined by exposing pieces of spinal cord (~20 segments) attached to the notochord to 50 μM NMDA. Ventral root activity was recorded using glass suction electrodes. Fictive activity varies markedly within and between experiments (Parker et al., 1998). To control for the former effect activity was only recorded when the activity had been stable for at least 2 h (see Parker et al., 1998). For analysis, 5 min of network activity was recorded in various conditions. As there is no way of standardizing the starting activity, which varies in frequency and quality for a given NMDA concentration across experiments (Parker et al., 1998), all values of fictive locomotion data were normalized to the mean of the control for each condition. This allowed the use of an ANOVA to test for significant differences (Valcu and Valcu, 2011).

The changes in Ringer Ca^2+^ were relatively small compared to that used in previous studies (e.g., Parker and Grillner, 1998) to try and limit effects to changes in activity-dependent synaptic plasticity. However, the potential for changes in properties other than short-term plasticity still existed and had to be checked. The EPSP half-width was measured to identify potential effects of altered Mg^2+^ levels on the NMDA component of the EPSP (Dale and Roberts, 1985; Dale and Grillner, 1986). Surface screening changes in excitability (Piccolino and Pignatelli, 1996) were avoided by adjusting Mg^2+^ levels to maintain divalent cation levels. Ringer-induced changes in cellular excitability were assessed from the number of spikes evoked by 100–400 ms depolarizing current pulses in motor neurons or unidentified cells from a current clamped potential of −70 mV. Global changes in spinal cord excitability were assessed from ventral root activity evoked by extracellular stimulation of the cell body region of the spinal cord approximately 5 segments rostral to the root recorded from (a single 1 ms stimulation at 1.5 times the threshold needed to evoke a single ventral root spike; see Cooke and Parker, 2009). Single stimuli were given 5 times at 0.2 Hz. The responses were rectified and integrated up to 150 ms after the stimulation and the amplitude of the averaged integrated trace was measured (see Hoffman and Parker, 2010).

### The network model

A simple model of the locomotor network was made in MATLAB using Simulink (Version 6.3 R14SP3). Two blocks were used, one representing a generic neuron and the other the synapse. A “motor neuron” served as an output measure.

#### The neuron block

The neuron model had a Na^+^, delayed rectified K^+^, leak K^+,^current. The resting potential for model neurons was −70 mV. Voltage dependent Na^+^ and K^+^ channels were modeled using Hodgkin-Huxley equations:

$$\begin{array}{lll} I_{Na} & = & \bar{G_{Na}}m^{3}h\left(V-E_{Na}\right)\\ I_{K} & = & \bar{G_{K}}n^{4}\left(V-E_{K}\right)\\ I_{leak} & = & G_{leak}\left(V-V_{rest}\right) \end{array}$$

in which *m, h*, and *n* were defined as:

$$\begin{array}{lll} \frac{dm}{dt} & = & \alpha_{m}\left(V\right)\left(1-m\right)-\beta_{m}\left(V\right)m\\ \frac{dh}{dt} & = & \alpha_{h}\left(V\right)\left(1-h\right)-\beta_{h}\left(V\right)h\\ \frac{dn}{dt} & = & \alpha_{n}\left(V\right)\left(1-n\right)-\beta_{n}\left(V\right)n \end{array}$$

These Rate Functions were defined as:

$$\begin{array}{lll} \alpha_{m}\left(V\right) & = & \frac{A_{\alpha_{m}}\left(V-B_{\alpha_{m}}\right)}{\left(1-e^{\left(B_{\alpha_{m}}-V\right)∕C_{\alpha_{m}}}\right)}\\ \beta_{m}\left(V\right) & = & \frac{A_{\beta_{m}}\left(B_{\beta_{m}}-V\right)}{\left(1-e^{\left(V-B_{\beta_{m}}\right)∕C_{\beta_{m}}}\right)}\\ \alpha_{h}\left(V\right) & = & \frac{A_{\alpha_{h}}\left(B_{\alpha_{h}}-V\right)}{\left(1-e^{\left(V-B_{\alpha_{h}}-V\right)∕C_{\alpha_{h}}}\right)}\\ \beta_{h}\left(V\right) & = & \frac{A_{\beta_{h}}}{\left(1+e^{\left(B_{\beta_{h}}-V\right)∕C_{\beta_{h}}}\right)}\\ \alpha_{n}\left(V\right) & = & \frac{A_{\alpha_{n}}\left(V-B_{\alpha_{n}}\right)}{\left(1-e^{\left(B_{\alpha_{n}}-V\right)∕C_{\alpha_{n}}}\right)}\\ \beta_{n}\left(V\right) & = & \frac{A_{\beta_{n}}\left(B_{\beta_{n}}-V\right)}{\left(1-e^{\left(V-B_{\beta_{n}}\right)∕C_{\beta_{n}}}\right)} \end{array}$$

The Rate Constants were:

$$\begin{array}{lll} A_{\alpha_{m}}\; & = & 0.2\times 10^{6},B_{\alpha_{m}}=-45\times 10^{-3},C_{\alpha_{m}}=1\times 10^{-3}\\ A_{\beta_{m}} & = & 0.06\times 10^{6},B_{\beta_{m}}=-54\times 10^{-3},C_{\beta_{m}}=20\times 10^{-3}\\ A_{\alpha_{h}} & = & 0.08\times 10^{6},B_{\alpha_{h}}=-45\times 10^{-3},C_{\alpha_{h}}=1\times 10^{-3}\\ A_{\beta_{h}} & = & 0.4\times 10^{3},B_{\beta_{h}}=-41\times 10^{-3},C_{\beta_{h}}=2\times 10^{-3}\\ A_{\alpha_{n}} & = & 0.02\times 10^{6},B_{\alpha_{n}}=-45\times 10^{-3},C_{\alpha_{n}}=0.8\times 10^{-3}\\ A_{\beta_{n}} & = & 0.005\times 10^{6},B_{\beta_{n}}=-35\times 10^{-3},C_{\beta_{n}}=0.4\times 10^{-3} \end{array}$$

#### The synapse block

The synapse block controlled the PSP sign, the initial amplitude, Facilitation and Depression (expressed in % per mV ms), and the Facilitation and Depression Recovery Rates (in % per ms). An action potential in the Neuron Block triggered the Synapse Block to generate a PSP. The PSP shape was determined by the PSP buffer. The Initial PSP Amplitude was set by a standard impulse response function *f*(*t*):

$$\begin{array}{l} PSP=\text{max}amplitude\times f\left(t\right) \end{array}$$

Where *f*(*t*) is:

$$\begin{array}{l} f(t)=\{\begin{array}{cc} e^{-\tau_{1}t}-e^{-\tau_{2}t}, & \;t-t_{1}<T\\ 0, & \;otherwise \end{array} \end{array}$$

This gave an exponential PSP decay. A synapse was defined by four parameters: the time when a spike was detected *t*_1_, duration *T*, rise time constant τ_1_, and decay time constantτ_2_. For the AMPA component, the parameters of the impulse response function were:

$$\begin{array}{l} \text{max}amplitude=0.004,\tau_{1}=0.1,\tau_{2}=0.3 \end{array}$$

NMDA-mediated components are slower than AMPA (Dale and Roberts, 1985; Dale and Grillner, 1986). The initial amplitude of the NMDA component was 25% of the AMPA component, and the rise and decay time twice that of the AMPA component. For the NMDA-mediated component, the parameters of the impulse response function were:

$$\begin{array}{l} \text{max}amplitude=0.001,\tau_{1}=0.05,\tau_{2}=0.15 \end{array}$$

A larger NMDA component was twice and a smaller NMDA component half the amplitude of the normal value.

This initial PSP was modified by activity-depednent plasticity. The final PSP was:

$$\begin{array}{l} PSP_{final}=PSP\times plasticity \end{array}$$

With depression or facilitation as:

$$\begin{array}{l} EPSP_{n}=PSP_{n}\times \int \left(1-Depression\times EPSP_{n-1}\right)dt \end{array}$$

$$\begin{array}{l} PSP_{final_{n}}=PSP_{n}\times \int_{1}^{10}\left(Faclitation\times EPSP_{n-1}\right)dt \end{array}$$

Depression and facilitation were adjusted by their recovery rates:

$$\begin{array}{l} depression=\int Depression\;Recovery\;Rate\times (1-depression)\\ \;-Depression)dt \end{array}$$

$$\begin{array}{l} facilitation=\int (Facilitation-Facilitation\;Recovery\;Rate\times \\ \;\left(facilitation-1\right))dt \end{array}$$

#### Network configuration

The network was built by connecting Neuron Blocks and Synapse Blocks to reflect different types of hemisegmental connections. The network had three interconnected excitatory interneurons, with one output motor neuron and in some cases one SiIN. The excitatory interneurons all connected to each other and to the motor neuron, with one excitatory interneuron receiving the driving input that activated the network. This was chosen to make the driving EIN spike at 5, 10, and 20 Hz. When feedback inhibition was added it connected to the driving excitatory interneuron.

## Results

### Analysis of synaptic connections

Previous analyses of the activity-dependent synaptic plasticity in the lamprey locomotor network lacked detail for connections between network interneurons (Parker and Grillner, 2000; Parker, 2003b). A more detailed analysis of these connections using data obtained since Parker (2003b) was thus performed here.

**Figure 1:**
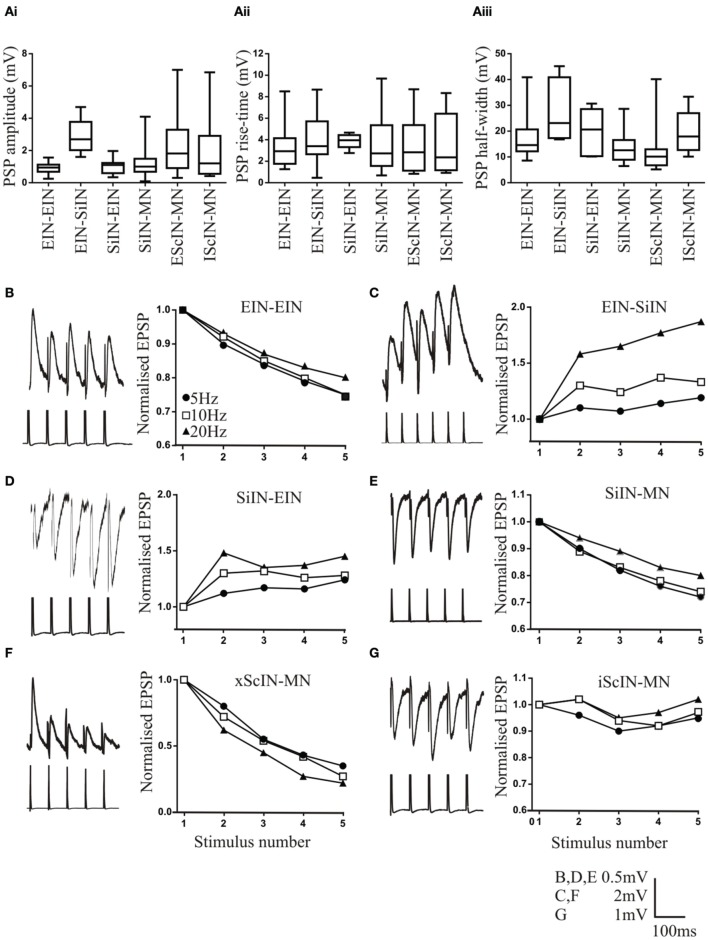
**Properties of connections between network interneurons**. The amplitude **(Ai)**, rise time **(Aii)**, and half-width **(Aiii)** at different connections. Symbols that show significant differences are not included on these graphs for clarity, but the differences are outlined in the text. Traces and graphs showing the activity-dependent plasticity at connections between excitatory interneurons (EIN; **B**), EINs inputs onto small ipsilateral inhibitory interneurons (SiIN; **C**), SiIN inputs onto EINs **(D)**, SiIN inputs onto motor neurons **(E)**, excitatory small crossing interneurons (xScIN) onto motor neurons **(F)**, and inhibitory small crossing interneurons (iScIN) onto motor neurons **(G)**. The lower part of the traces are the presynaptic action potentials, which in some cases have been clipped. Error bars are not shown for clarity, but SEM values are included in the text.

EINs monosynaptically connect to each other to form a potential excitatory hemisegmental group (Parker and Grillner, 2000). The sample size of this connection has increased from *n* = 4 (Parker and Grillner, 2000) to *n* = 33. The connection had a mean amplitude of 0.93 ± 0.57 mV (*n* = 33; Figure 1Ai), which was significantly smaller than the EIN input to motor neurons (Parker, 2003a), a rise time of 3.1 ± 2.04 ms (*n* = 26; Figure 1Aii), and a half-width of 17.2 ± 10.2 ms (*n* = 26; Figure 1Aiii), which was significantly longer than the EPSP half-width in motor neurons (Parker, 2003a). The connection usually depressed over spike trains (*n* = 27 of 33; Figure 1B). The depression reached 75 ± 4% of control by the 5th spike in the train at 5 Hz, 75 ± 6% at 10 Hz, and 80 ± 5% of control at 20 Hz (*p* < 0.05). The extent of depression over the spike train for the most part matched that of EIN inputs to motor neurons (Parker, 2003a). In addition to these depressing connections, three connections were unchanged and three facilitated (data not shown).

EIN to EIN connections exhibited less variability than EIN inputs to motor neurons. This related to the initial EPSP amplitude (EIN-EIN range 0.25–1.42 mV; motor neuron range 0.32–4.2 mV), and the activity-dependent plasticity over spike trains. For EIN-EIN connections, 82% depressed, 9% facilitated, and 9% were unchanged, whereas for EIN inputs to motor neuron, 46% depressed, 27% facilitated, 12% unchanged (Parker, 2003a; *p* < 0.05, Chi square).

Paired recordings previously failed to find reciprocal connections between the EINs (Parker and Grillner, 2000), and while the sample size has increased eight-fold no reciprocal connections were found.

The EINs connect to small ipsilateral inhibitory interneurons (SiIN; Parker, 2003b). The sample size of this connection has increased slightly (from *n* = 7 Parker, 2003b to *n* = 12). The mean amplitude of the connection was 2.9 ± 1.04 mV (range 0.7–4.1 mV; *n* = 12; Figure 1Ai), the rise time 4.4 ± 2.54 ms (*n* = 8; Figure 1Aii), and the half-width 27.4 ±10.32 ms (*n* = 7; Figure 1Aiii). Both the EPSP amplitude and half-width were significantly larger than that at EIN to EIN or EIN to motor neuron connections (*p* < 0.05). The connection consistently facilitated (*n* = 11 of 12, Figure 1C; the remaining connection was unchanged, data not shown). Significant facilitation occurred by the 5th spike in the train at 5 Hz (119 ± 8.5%), 10 Hz (133 ± 8%), and 20 Hz (187 ± 17%; Figure 1C). This contrasts the previous study (Parker, 2003b) where significant facilitation only occurred at 20 Hz. However, facilitation here was significantly greater at 20 Hz than at 5 and 10 Hz.

The SiINs provide feedback inhibition to the EINs (Parker, 2003b). The sample size of this connection has increased from *n* = 3 to *n* = 15. The connection had a mean amplitude of −0.97 ±0.46 mV (*n* = 15; Figure 1Ai), a rise time of 3.9 ± 0.6 s (*n* = 9; Figure 1Aii), and a half-width of 19.8 ± 8.2 ms (*n* = 7; Figure 1Aiii). The connection typically facilitated (*n* = 11; 1 connection depressed and 3 were unchanged; Figure 1D). The facilitation was significant by the 5th spike in the train at 5 Hz (124 ± 4%), 10 Hz (128 ± 4%), and 20 Hz (145 ± 7%; Figure 1D). The facilitation at 20 Hz was significantly greater than at 5 or 10 Hz. No reciprocal connections have been found between the SiINs and EINs.

The final hemisegmental connection examined was the SiIN-mediated feedforward inhibitory input to motor neurons (Buchanan and Grillner, 1988). A large sample size of these connections has been obtained (*n* = 95). The connection had a mean amplitude of −1.16±0.78 mV (*n* = 95; Figure 1Ai), a rise time of 3.4 ± 3.43 (*n* = 47; Figure 1Aii), and a half width of 11.95 ± 5.93 ms (*n* = 32; Figure 1Aiii). These values were not significantly different to EIN-evoked EPSPs in motor neurons (Parker, 2003a). Depression was the commonest effect over spike trains (*n* = 45 of 92; Figure 1E): facilitation occurred in 35 connections, 3 were biphasic (facilitation followed by depression), and 12 connections were unchanged (data not shown). The relative proportions of the different types of plasticity did not differ to EIN inputs to motor neurons (*p* > 0.05; Chi square; Parker, 2003a). The level of depression by the 5th spike in the train was 72 ± 10% of control at 5 Hz, 74 ± 4% at 10 Hz, and 80 ± 2% at 20 Hz: these values were all significant, but the level of depression did not differ significantly at the different stimulation frequencies (Figure 1E). Facilitation by the 5th spike in the train was 143 ± 20% at 5 Hz, 134 ± 4% at 10 Hz; and 127 ± 6% at 20 Hz (data not shown). The level of facilitation was significant at each frequency, but did not differ at the different frequencies.

In addition to hemisegmental connections, the sample size of connections between excitatory and inhibitory small crossing interneurons (SCiN; Parker and Grillner, 2000) has increased [excitatory ScIN (*n* = 47), inhibitory ScIN (*n* = 17)]. Inputs have only been shown onto motor neurons, and thus the connectivity of these cells with the opposite hemisegment remains a major gap in understanding network connectivity (see Parker, 2006). Excitatory ScINs had a mean amplitude of 2.2 ± 1.99 mV (*n* = 47; Figure 1Ai), a rise time of 3.3 ± 6.23 ms (*n* = 27; Figure 1Aii), and a half-width of 11.3 ± 11.03 ms (*n* = 23; Figure 1Aiii). The connections usually depressed strongly over spike trains [*n* = 27 of 32; 5 Hz (35 ± 7%), 10 Hz (27 ± 4%) and 20 Hz (22 ± 4%); Figure 1F]: the remaining connections were unchanged. The inhibitory ScINs had a mean amplitude of −1.9 ± 2.89 mV (*n* = 17; Figure 1Ai), a rise time of 3.2 ± 3.11 ms (*n* = 12; Figure 1Aii), and half-width of 19.9 ± 7.2 ms (*n* = 9; Figure 1Aiii). The input was usually unchanged over spike trains (*n* = 14 of 17; Figure 1G): the other three connections depressed (87 ± 4%; data not shown).

The data highlights the synapse-specific properties of connections. For single PSPs the EIN to SiIN amplitude was significantly greater than all other connections, with the exception of inhibitory ScIN inputs to motor neurons. The variance of inhibitory ScIN inputs was also significantly greater than that of other connections (*p* < 0.05, *F*-test; the variability of other connections did not differ). Rise times did not differ between connections. The EIN-SiIN half-width was significantly greater than EIN-EIN, SiIN-motor neuron, and excitatory ScIN to motor neuron connections, with the latter two connections having significantly smaller half-widths than the other connections, and the EIN-EIN EPSP half-width was significantly longer than EIN to motor neuron half-width. The type of activity-dependent plasticity shown was synapse-specific, but levels of facilitation and depression were generally similar between connections, with the exception of excitatory ScINs which showed marked depression. The proportion of different forms of activity-dependent plasticity did differ: EIN (Parker, 2003a) and SiIN connections to motor neuron showed significantly greater variability than connections between interneurons (*p* < 0.05, Chi Square).

### Cellular and synaptic effects of changing ringer Ca^2+^ levels

Linking activity-dependent synaptic properties to network activity requires that synaptic properties are manipulated. This is typically done with low and high Ca^2+^ Ringer (Zucker and Regehr, 2002). The effects of these Ringers had to be examined to determine how synaptic properties were affected. This was done on EIN-evoked EPSPs to motor neurons as this connection is found readily and is relatively stable. This, however, rests on the assumption that similar effects occur at other connections.

**Figure 2:**
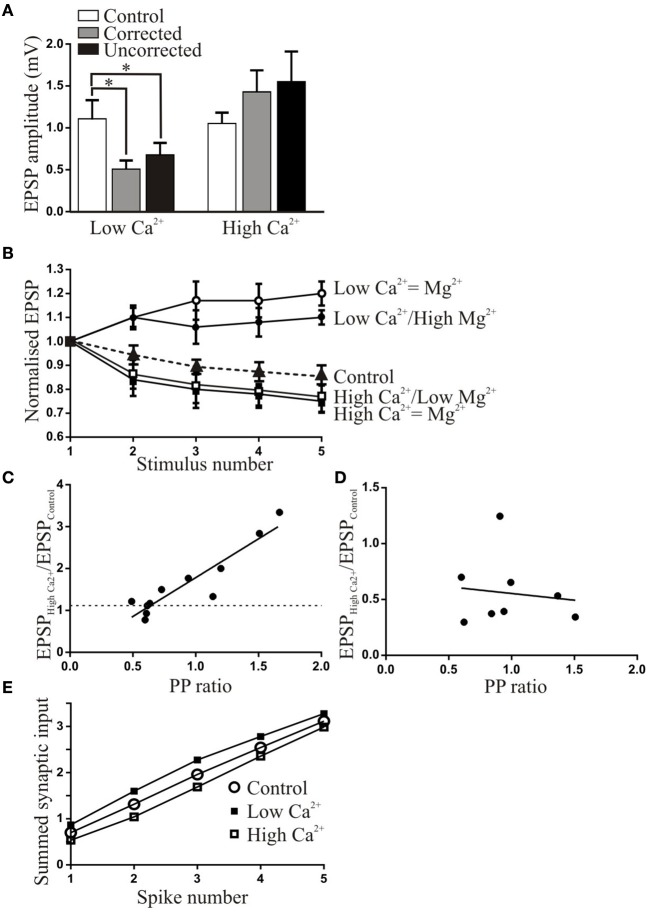
**The effects of altered Ringer Ca^2+^ solutions on synaptic inputs from EINs to motor neurons**. **(A)** The effect of corrected and uncorrected high and low Ca^2+^ Ringer on the amplitude of low frequency-evoked EPSPs. On this and subsequent graphs the asterisk shows statistically significant differences. **(B)** The activity-dependent plasticity in corrected and uncorrected high and low Ca^2+^ Ringer. The initial EPSP has been normalized in each case to show the activity-dependent changes. **(C)** The relationship between the PP ratio (EPSP_2_/EPSP_1_) and the normalized change in the EPSP in uncorrected high Ca^2+^ Ringer. The dashed line shows the 110% of control value. **(D)** Graph showing the lack of relationship between the PP ratio and the change in the EPSP in corrected low Ca^2+^ Ringer. **(E)** Graph showing the summed EPSPs over the spike train in control and low and high Ca^2+^ Ringer. The changes in the initial EPSP amplitude lead to different initial starting points and an increased or decreased summed input over the initial part of the spike train, but depression and facilitation lead to final summed inputs matching that in control.

Corrected (*n* = 7 of 8) or uncorrected (*n* = 6 of 6) low Ca^2+^ Ringer significantly reduced the amplitude of low frequency-evoked EPSPs (Figure 2A). High Ca^2+^ Ringer had more variable effects, with no significant change when the data was grouped (Figure 2A; in individual experiments there was a significant increase in the EPSP amplitude in 4 of 8 connections in corrected Ringer and in 8 of 11 connections in uncorrected Ringer). When all connections were grouped low Ca^2+^ Ringer significantly reduced depression or evoked facilitation (facilitation was usually evoked at 20 Hz, reduced depression at 5 or 10 Hz; Figure 2B). While the effect was less pronounced than that of low Ca^2+^ Ringer, high Ca^2+^ Ringer significantly increased depression by the 5th spike in the train (Figure 2B).

The variable effects of high Ca^2+^ Ringer may relate to the initial release probability of connections (Parker, 2003a): there could be less scope for an increase in the amplitude when the release probability was relatively high. This was supported by the significant positive correlation between the change in EPSP amplitude and paired pulse (PP) ratio (*r*^2^ = 0.73; Figure 2C). Connections with higher release probabilities (lower PP ratio; see Parker, 2015) may have been less affected because they were closer to maximal efficacy (release probability-independent effects could also contribute; Spencer et al., 1989). There was no significant relationship between the PP ratio and the EPSP reduction in low Ca^2+^ Ringer (*r*^2^ = 0.01; Figure 2D), suggesting that a reduction could occur irrespective of the initial properties of the connection.

While the Ringer manipulations had the intended effect of altering activity-dependent synaptic properties, the changes in the initial EPSP amplitude will influence the overall synaptic drive. This is shown in Figure 2E where the successive EPSPs are summed. In high Ca^2+^ even though the initial EPSP amplitude was not significantly increased there was a larger summed input over most of the spike train compared to control, and for low Ca^2+^ there was a reduced input. The development of facilitation and depression however meant that by the end of the spike train the effects matched that in control.

In addition to synaptic effects, changing Ringer Ca^2+^ could also affect excitability through surface screening (Piccolino and Pignatelli, 1996) or changes in the amplitude of the calcium-dependent slow afterhyperpolarization (sAHP). Corrected divalent cation Ringer can control for surface screening, but adds the complication of potential Mg^2+^- dependent changes in NMDA conductance. These potential effects had to be examined as they could also contribute to any network effects of the Ringer changes.

**Figure 3:**
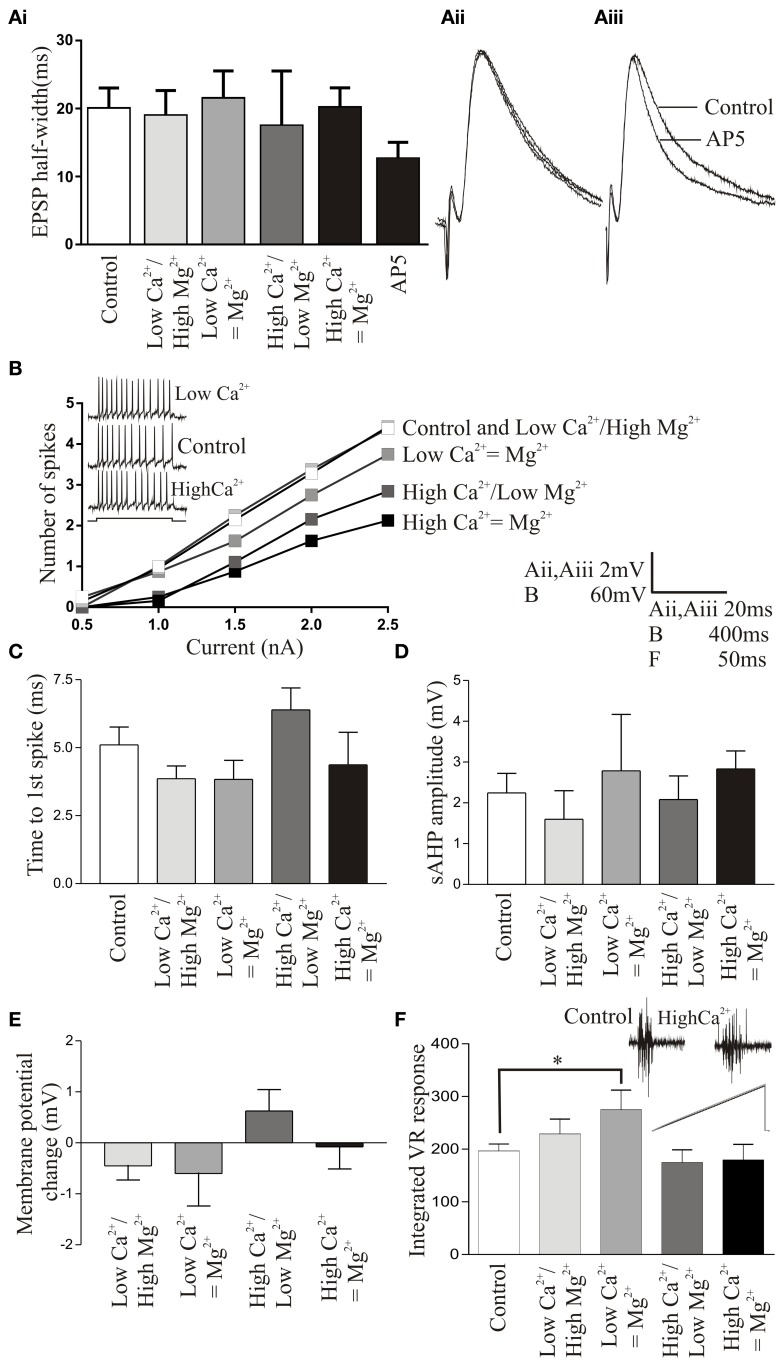
**(Ai)** Graph showing the lack of change in the EPSP half-width in control and altered Ringer solutions. All recordings are made from unidentified cells or motor neurons. **(Aii)** Overlaid traces of averaged EPSPs in normal, high Ca^2+^/low Mg^2+^, and low Ca^2+^/high Mg^2+^ Ringer. **(Aiii)** Traces showing averaged EPSPs in control and after application of AP5 (100 μM). **(B)** Graph showing the number of spikes evoked in different Ringer solutions in response to current pulses of 0.5–2.5 nA. Error bars are not shown for clarity, but there were no significant differences in the number of spikes. The inset shows the spiking evoked in response to a 400 ms current pulse in normal Ringer (middle trace), low Ca^2+^ Ringer (top trace), and high Ca^2+^ Ringer (lower trace). **(C)** Graph showing the lack of a change in the time to the first spike in different Ringer solutions. **(D)** Graph showing the lack of a significant effect on the slow after hyperpolarization in the different Ringer solutions. **(E)** Graph showing the change in resting membrane potential (RMP) in the different Ringer solutions. **(F)** Graph showing the integrated ventral root response evoked by a single stimulation of the cell body region of the spinal cord. The inset shows traces in control (left) and corrected high Ca^2+^ Ringer (right). The lower part of the trace shows overlaid rectified and integrated responses (gray line is control).

The effects of the different Ringer solutions on the NMDA component of the EPSP was examined by measuring the half-width of EPSPs evoked by reticulospinal axons (*n* = 12; (Dale, 1986)). In these experiments we saw no significant effect on the EPSP half-width in high Mg^2+^ /low Ca^2+^ (*n* = 9) or low Mg^2+^/high Ca^2+^ Ringer (*n* = 12) suggesting against a marked change in the NMDA component of the EPSP (Figures 3Ai,Aii). There was again some variability: in 3 connections there was a marked reduction in the EPSP half-width in high Mg^2+^ /low Ca^2+^ Ringer. Whether this reflects some variability in NMDA responses in different experiments, for example in NMDA subunit composition (Kuner and Schoepfer, 1996) is not known at present. Mg^2+^ does affect NMDA responses in lamprey, but this was shown by going from zero to normal Mg^2+^ levels (Dale, 1986; Buchanan et al., 1987), while here Mg^2+^ was always present which may account for the overall lack of significant effect. To ensure that the lack of effect was not due to the absence of an NMDA component on the EPSPs we examined, we tested the effects of AP5 (100 μM) after the different Mg^2+^ Ringers had been examined (*n* = 7). While the different Ringer solutions did not affect the half-width, AP5 resulted in a significant reduction of the EPSP half width in these connections (*p* < 0.05; Figures 3Ai,Aiii).

When depolarizing current pulses were injected into cells to evoke trains of spikes, corrected (*n* = 10) or uncorrected (*n* = 10) low Ca^2+^ Ringer did not significantly affect spiking over the current pulse compared to control: corrected (*n* = 7 of 10) or uncorrected (*n* = 4 of 5) high Ca^2+^ Ringer usually reduced excitability (Figure 3B), but this was not statistically significant. There was also no significant difference in the time of the first spike compared to control in any Ringer (Figure 3C). The sAHP was affected in ways that would be expected, with low Ca^2+^ Ringer reducing the amplitude and high Ca^2+^ increasing it (Figure 3D), but the changes were again not statistically significant. Although excitability was assessed from a current clamped potential of -70 mV to avoid changes due to differences in resting membrane potential (RMP), the RMP was also monitored: there was no significant change in RMP in the different Ringer solutions (Figure 3E).

While these effects were not significant in single cells, there were effects of the different Ringer solutions and variability between cells. It could be argued that while these various effects were statistically non-significant that they could individually or in combination have had significant network effects. Global effects were examined using spinal cord stimulation-evoked activity recorded from the ventral root (Figure 3F; see Materials and Methods). In most cases the different Ringers had no significant effect on ventral root activity, although there was a significant increase in excitability in uncorrected low Ca^2+^ Ringer compared to control.

### Analyses of altered Ca^2+^ ringers on fictive activity in intact spinal cords

**Figure 4:**
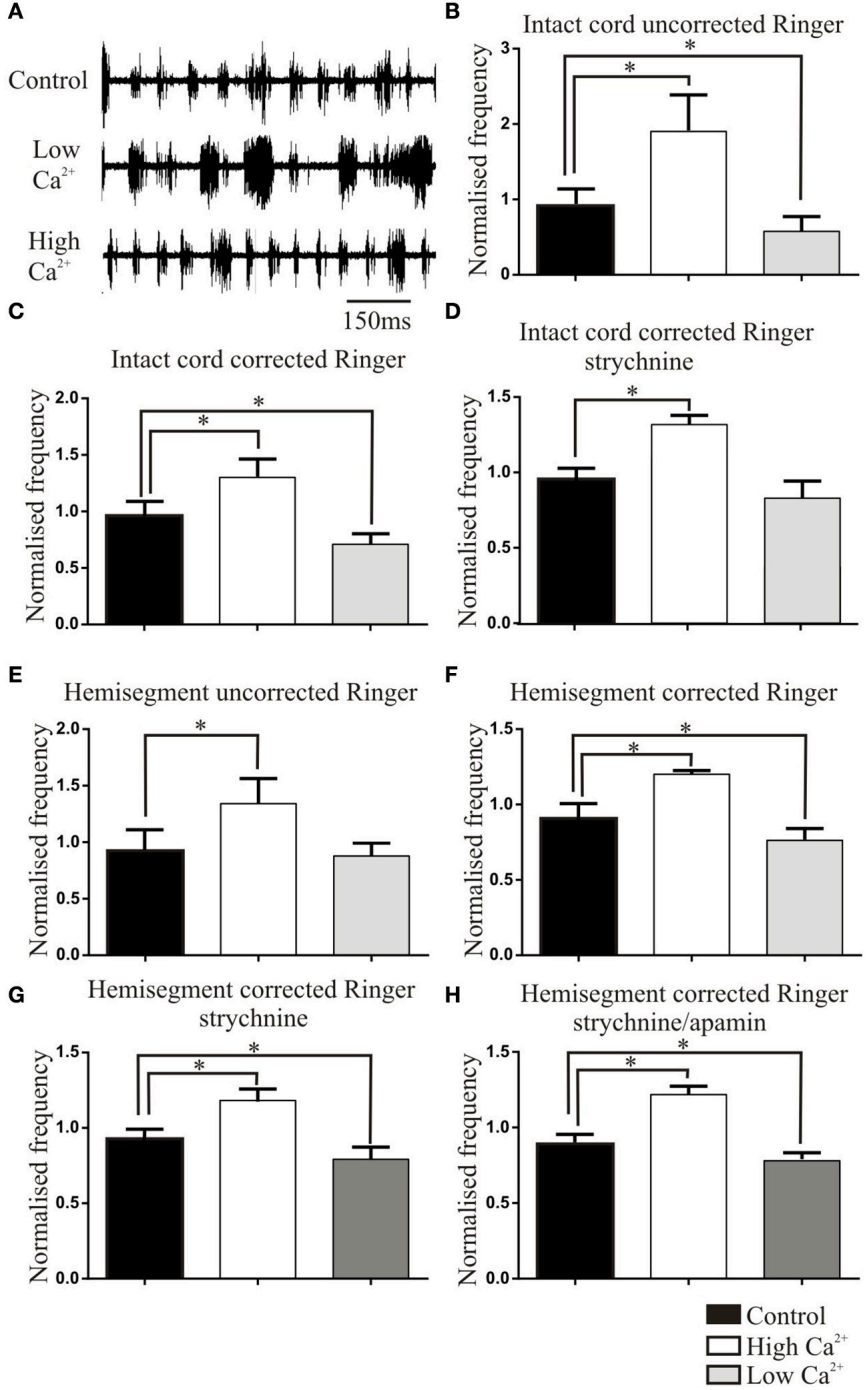
**(A)** Traces showing the effects of high and low Ca^2+^ Ringer on NMDA (50 μM)-evoked ventral root activity. **(B)** Graphs showing effects in the intact spinal cord of uncorrected Ringer **(B)**, corrected Ringer **(C)**, corrected Ringer in strychnine (1 μM; **D**). Graphs showing the effects in the hemicord of uncorrected Ringer solutions **(E)**, corrected Ringer solutions **(F)**, corrected Ringer in strychnine (1 μM; **G**), and corrected Ringer in strychnine (1 μM) and apamin (2 μM; **H**). Note that effects were normalized here to the mean of the control values (see Materials and Methods) to allow statistical comparisons. The normalized control value thus is not necessarily 1 and has an SEM.

In an attempt to relate changes in activity-dependent synaptic properties to the patterning of network activity, the effects of altered Ringer Ca^2+^ solutions were examined on NMDA (50 μM)-evoked fictive locomotion. The burst frequency was reduced in uncorrected low Ca^2+^ Ringer and increased in high Ca^2+^ Ringer (*n* = 22; Figure 4A). These changes were not significant when the raw data was used (*p* > 0.05, *t*-test), but as this could have reflected the wide variability of initial values in different cords (see Parker et al., 1998; Zhang and Grillner, 2000), values were normalized to the mean control value. This resulted in high and low Ca^2+^ values that were significantly different to control (*n* = 22, *p* < 0.05; Figure 4B).

Corrected Mg^2+^ Ringer was used to avoid potential surface screening effects on excitability (Figure 4C). The normalized low and high Ca^2+^ corrected Ringer frequencies were still significantly lower and higher, respectively, than control (*n* = 17, *p* < 0.05). However, the effect of corrected high Ca^2+^ Ringer was significantly reduced compared to the uncorrected Ringer effect (*p* < 0.05). This could suggest an influence of excitability changes in uncorrected Ringer or Mg^2+^-dependent effects on NMDA conductances in corrected Ringer (although reduced Mg^2+^ in high Ca^2+^ Ringer should increase the frequency; Brodin and Grillner, 1986), or the variability of effects between different cords (see Parker et al., 1998).

The changes in frequency in high and low Ca^2+^ Ringer could reflect changes at several sites: depressing EIN connections, the facilitating hemisegmental inhibitory feedback circuit (EIN-SiIN-EIN; Parker, 2003b), or crossing connections that determine when the previously silent side escapes from inhibition (both crossing inhibitory and excitatory inputs; Buchanan, 2001; Parker, 2006). The involvement of inhibitory connections was examined by blocking glycinergic inhibition with strychnine (1 μm, *n* = 17). This either disrupted activity, preventing further analysis (*n* = 7), or evoked slightly faster synchronous or independent activity on the two sides. In strychnine the increase in frequency in corrected high Ca^2+^ Ringer persisted, but the significant effects of low Ca^2+^ Ringer were abolished (*n* = 10; Figure 4D; uncorrected Ringer solutions were not tested with strychnine).

While these effects were generally consistent with changes in activity-dependent synaptic properties, the link was complicated by the various synapses that could be affected. To limit this, effects were examined in hemisected spinal cords to remove crossing inputs: these represent 50% of the neurons in the network and remain poorly understood (Parker, 2006). The depressing excitatory and facilitating inhibitory feedback group (EIN-SiIN-EIN) could both act as burst terminating factors by gradually reducing excitation and increasing inhibition during a hemisegmental burst. Corrected or uncorrected high Ca^2+^ Ringer increased the frequency of hemisegmental network activity (*n* = 16). Uncorrected low Ca^2+^ Ringer did not significantly affect the frequency (*p* > 0.05, Figure 4E), but the effect was significant in corrected Ringer (*p* < 0.05; Figure 4F). In corrected Ringer in strychnine (1 μM) to block the facilitating feedback inhibitory circuit, the changes in frequency in low and high Ca^2+^ Ringer persisted (*n* = 11; Figure 4G), an effect that suggests a dominant role for the depressing EIN component in patterning hemisegmental activity.

While corrected Ringer effects should remove an influence of surface screening, it would not control for changes of the Ca^2+^-dependent sAHP (Hill et al., 1992). The different Ca^2+^ Ringers were thus examined in strychnine and apamin (2 μM; Hill et al., 1992) to block the sAHP and thus remove its potential influence. The effects of low and high Ca^2+^ corrected Ringer persisted (*n* = 6; Figure 4H), suggesting against an influence of the sAHP changes. This successive peeling back effects from the intact to hemisected cord with inhibition and the sAHP blocked support a role for activity-dependent depression of the EIN group in patterning network activity.

### Modeling activity-dependent effects

While these analyses suggest activity-dependent synaptic plasticity could influence network activity, even a hemisegment in strychnine and apamin has the potential for effects other than the targeted synaptic changes to influence network activity. A simple network model in Matlab (see also Dale, 1995; Nadim et al., 1999; Tabak et al., 2001) that used experimental values for activity-dependent plasticity was used to examine how the activity-dependent plasticity identified above could influence the patterning of network outputs.

**Figure 5:**
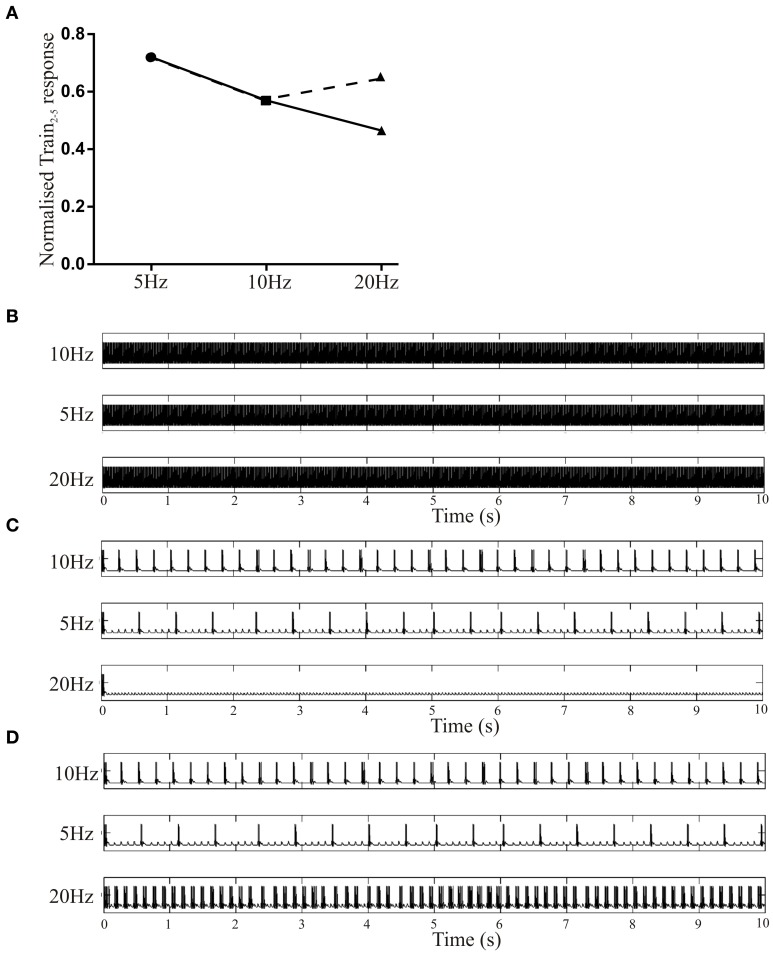
**(A)** Simulation result showing the normalized Train2–5 EPSP amplitudes at different frequencies at a single synapse. The solid line shows the effects with a fixed recovery depression rate (D_Rec_) of 0.32. The dashed line shows the effects of increasing this to 0.9. **(B)** Simulated network output when there was no activity-dependent plasticity. **(C)** Simulated network output when Depression (1.2) was added and D_Rec_ was 0.32. **(D)** Simulated network with a Depression of 1.2 and a D_Rec_ of 0.9.

Depression due to depletion is balanced by replenishment (see Zucker and Regehr, 2002). At the EIN to MN synapse replenishment is activity-dependent (Parker, 2000), although the actual depression recovery rate is unknown. Simulated effects at a single synapse were thus adjusted to match experimental effects. (Parker, 2000). Changes in the EPSP were examined over the 2nd to 5th EPSPs normalized to the amplitude of the initial EPSP. With an initial amplitude of 3 mV, Depression (Dep) of 1.2, and a Depression Recovery Rate (D_Rec_) of 0.32, depression increased linearly with stimulation frequency, which contrasts experiments where depression is reduced at 20 Hz (Parker, 2000; Figure 5A). When D_Rec_ was increased to 0.9 depression was reduced at 20 Hz. These depression effects at different frequencies matched the experimental data on the depression of EIN inputs to motor neurons and other EIN connections (Parker, 2000, 2003a).

With no activity-dependent depression and the driving EIN spiking at 5, 10, or 20 Hz (initial EPSP amplitude 3 mV) network activity was tonic (Figure 5B). With Depression (1.2) but no D_Rec_ activity fell silent (data not shown). When D_Rec_ (0.32) was added bursting occurred when the driving EIN spiked at 5 and 10 Hz, suggesting that depression can act as a burst terminating factor (Figure 5C). There was no bursting when the driving EIN spiked at 20 Hz unless D_Rec_ was increased to 0.9 (Figure 5D), supporting the need for activity-dependent replenishment (Parker, 2000).

**Figure 6:**
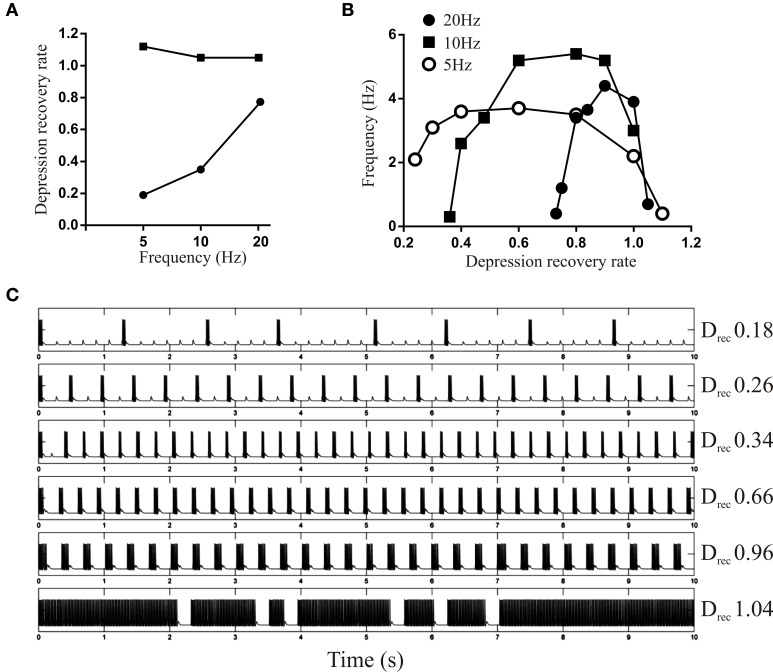
**(A)** Graph showing the upper and lower limit of D_Rec_ when the Depression was held fixed at 1.2. Note that the upper limit is the same at all frequencies, but the lower limit increases in a frequency-dependent manner. **(B)** Graph showing the burst frequency of the network output for a driven frequency of 5, 10, and 20 Hz over a range of D_Rec_. **(C)** Traces showing network activity evoked from a driven frequency of 5 Hz for different values of D_Rec_.

The influence of depression on bursting was examined using a fixed Depression or D_Rec_ level while varying the other parameter. With Depression fixed at 1.2, the upper limit of D_Rec_ was almost constant when the driving EIN spiked at 5, 10, or 20 Hz, while the lower limit increased approximately linearly with frequency (Figure 6A). The region between the upper and lower limits is the range of D_Rec_ values that could maintain some form of rhythmic network activity: above the upper limit activity was tonic and below the lower limit the network fell silent. The active range narrowed as the frequency increased. At all frequencies there was an inverted U shape between D_Rec_ and the burst frequency (Figures 6B,C), presumably because when D_Rec_ was low depression summed over successive bursts causing activity to fail, and when it was too high depression would not develop and burst termination in the model was compromised.

**Figure 7:**
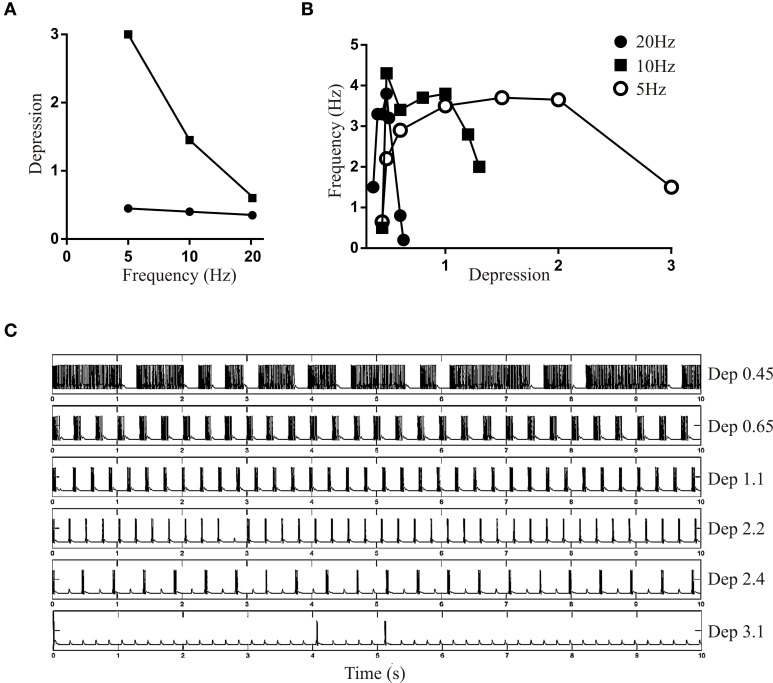
**(A)** Graph showing the upper and lower limit of Depression when D_Rec_ was held fixed at 0.4. **(B)** Graph showing the frequency of network bursting at 5, 10, and 20 Hz over a range of Depression values. **(C)** Traces showing the network activity over a range of Depression values when the network was driven at 5 Hz.

With D_Rec_ fixed (here at a value of 0.4 to be between the lower and higher frequency values above) and Depression was varied the lower limit of the Depression that could maintain bursting was nearly constant, but there was a frequency-dependent decrease in the upper limit (Figure 7A). The region between the upper and lower limits again represented the range over which Depression could maintain network activity. The active range narrowed as the frequency increased. Depression could presumably be greater with lower frequencies (Figure 7B) because there was more time for recovery between spikes. As with D_Rec_, there was an inverted U shape for the relationship between Depression and burst frequency (Figures 7B,C).

**Figure 8:**
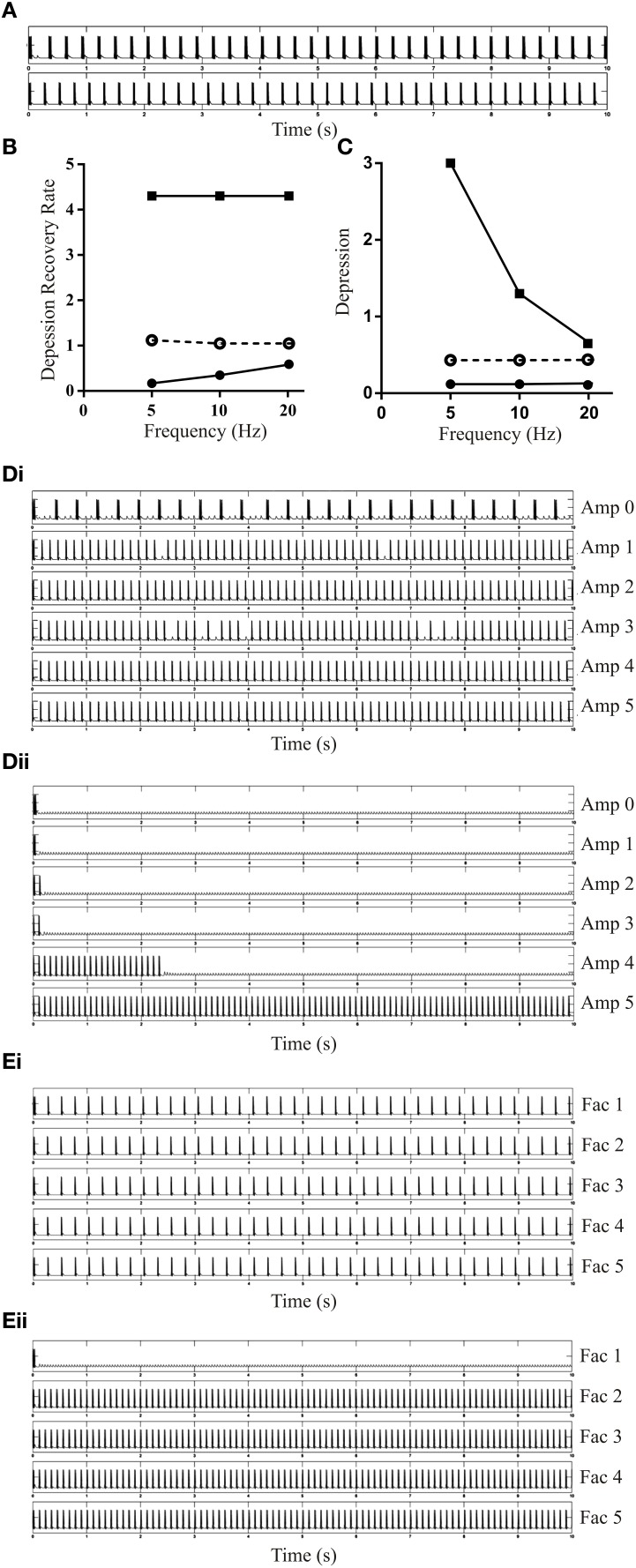
**The effects of adding feedback inhibition on simulated network activity**. **(A)** The upper trace shows a network with no feedback inhibition, and the lower trace when feedback inhibition added (EIN Depression 1.2, D_Rec_ 0.4). **(B)** The lower and upper limits of D_Rec_ at different frequencies when Depression wass fixed at 1.2 with inhibitory feedback added to the network. The dashed line shows the upper value when no feedback inhibition was present. **(C)** The lower and upper limits of Depression (D_Rec_ 0.4) in the inhibitory feedback network. The dashed line shows the upper value when feedback inhibition was absent. Note that feedback inhibition increased the upper limit of D_Rec_ **(B)** and reduced the lower limit of Depression **(C)**. **(Di)** The output of the network with different amplitudes of the feedback IPSP (EIN Depression 1.2 and D_Rec_ 0.4). The driven frequency in this example was 10 Hz. **(Dii)** The MN output of the network with different Initial Amplitude of the feedback SiIN PSP. The EINs were plastic with Depression 1.2 and D_Rec_ 0.4. The driven frequency in this example was 20 Hz. **(Ei)** The bursting activity of the network with facilitated inhibitory feedback and different Facilitation levels. The parameters for EINs are unchanged from **(Di)**. The Initial Amplitude for the feedback inhibition was −1 mV and driven frequency was 5 Hz. **(Eii)** The bursting activity of the network with facilitated inhibitory feedback when the driven frequency was 20 Hz.

When inhibition was added to the EINs (Depression 1.2, D_Rec_ 0.4) to simulate SiIN-mediated feedback inhibition (initial amplitude of −1 mV with no plasticity), the burst frequency increased slightly (Figure 8A). However, a more dramatic effect occurred on the active range of the EIN Depression and D_Rec_, which increased three-fold with feedback inhibition (Figures 8B,C). This influenced the ability to evoke bursting with different values of D_Rec_. With a driven frequency of 5 or 10 Hz, as the amplitude of the feedback inhibitory input increased the bursts became shorter and the burst frequency increased (see Figure 8Di for 10 Hz activity). With a driven frequency of 20 Hz bursting did not develop with Dep of 1.2 and D_Rec_ of 0.4 as the recovery rate fell outside the active range (see Figure 5A). However, as the IPSP amplitude and thus the active range of depression increased, bursting activity developed (Figure 8Dii).

When Facilitation was added to the feedback inhibition to match the dominant experimental feature of this connection, the network activity was not affected with driven frequencies of 5 Hz (Figure 8Ei) or 10 Hz (data not shown), but at 20 Hz bursting activity, which was absent with no inhibition, developed as the strength of the inhibitory feedback increased (Figure 8Eii). This was similar to the effect seen with a tonic increase in IPSP amplitude but because of facilitation it occurred from smaller initial IPSP amplitudes.

## Discussion

While it has been suggested as a potential network building-block (e.g., Selverston, 1980), the influence of activity-dependent plasticity in neuronal networks is not well understood (O'Donovan and Rinzel, 1997; Nadim and Manor, 2000; Thomson, 2000a,b). This reflects the general difficulty of characterizing cellular and synaptic properties and then linking them to network activity, with the added complication of the potential circular interaction between network activity and activity-dependent synaptic processes. This study attempted to provide evidence that short-term synaptic plasticity can influence the patterning of locomotor network activity.

The analysis of interneuron connectivity performed here does not address the gaps that exist in the network organization (Parker, 2006), but characterizes the properties of previously identified connections between network interneurons (Parker, 2003b). The data strengthens the case for a predominantly depressing excitatory group of EINs connecting to other EINs and motor neurons (Parker, 2003a), and a feedback inhibitory group consisting of facilitating EIN and SiIN connections. The data also highlights synapse-specific properties of the connections. One aspect of note that has come out of the increased sample size of connections is that connections onto motor neuron show significantly greater variability than connections between interneurons. Sample sizes of interneuron to interneuron connections remain relatively low compared to inputs to motor neurons and this conclusion may change if sample sizes increased, but currently this suggests that the variability of the network output (e.g., Parker et al., 1998; Zhang and Grillner, 2000) may reflect the variability of the input to motor neurons rather than variability within the rhythm generating circuit.

While connectivity within a hemisegment is better understood than connectivity between hemisegments (see Parker, 2006), important details are still lacking. The obvious one is that little is known of the connectivity between the EINs. The only previous study that showed connectivity between these cells, in a very small sample (*n* = 4), was Parker and Grillner (2000). A subsequent review of the locomotor network connectivity claimed that the connection ratio between the EINs is ~10% (Grillner et al., 2005), but there is no reference to where this value comes from or how it was obtained (Parker and Grillner, 2000 is not cited as providing the evidence for this connection, but in any case this paper does not mention a connectivity ratio). No reciprocal connections have been found between these cells in this or the previous analysis of the connection (cf. Li et al., 2009 in tadpole), which suggests against a densely interconnected population of cells. Using a binomial model, the number of connections found here (*N* = 33 with no reciprocal connections) suggests a very low connection probability, *p*, of 0.05 that would have been obtained in a population of >300 paired recordings from EINs (for single connections Np(1-p), for reciprocal connections Np^2^, and for a lack of connection between pairs of EIN N(1-p)^2^; see Song et al. (2005). As various factors can influence success in recording from pairs of EINs and thus of finding single or reciprocal connections (e.g., marked differences in the ease of access of the cord in different experiments; the location of the electrode in an EIN, and thus the ease with which the electrode could be displaced when looking for another cell), this is a very tentative measure. However, from the data available at present there is no support for reciprocal connections between pairs of EINs. Rather than a reciprocally connected interneuron population, the network excitatory drive could instead reflect feedforward excitatory interactions (e.g., Bienenstock, 1995), where different layers of EINs, possibly reflecting different functional EIN sub-groups (Parker, 2003a), are activated by divergent and convergent connections from the preceding layer, with some or all of these layers also connecting to motor neurons (the possibility also exists for polysynaptic feedback connections). It could be speculated that the longer synaptic integration time offered by the longer half-width of EIN-EIN EPSPs may facilitate the summing of activity in an organization of this sort.

The changes in synaptic inputs were evoked by trains of five spikes. This is the upper limit of the number of spikes evoked during network activity (Buchanan and Cohen, 1982; Buchanan and Kasicki, 1995). Effects have only been studied using this number of spikes, and it could be argued that the effects here only reflect changes at an extreme of the spiking range. However, a large proportion of the final change in synaptic strength occurred over the first two spikes, especially for the facilitating connections, suggesting that activity-dependent synaptic plasticity could still be significant with shorter spike trains.

Linking any cellular or synaptic effect to network activity is conceptually difficult, and we cannot at this stage make any claim to providing direct links between activity-dependent synaptic changes and network activity. The changes in burst frequency in high and low Ca^2+^ Ringer do support a role for activity-dependent plasticity in patterning network activity. While changing Ca^2+^ is currently the only approach open to this analysis, it is a crude manipulation that has the potential for non-synaptic effects. However, while synaptic depression and facilitation were evoked in the ways expected at EIN to motor neuron synapses (admittedly requiring the assumption that similar changes occur at other synapses), the different Ringers evoked no significant changes in the RMP or cellular excitability. From Hille (1968) the changes in cation levels in the uncorrected Ringer used here should change the spike threshold by 2–3 mV, suggesting that the relatively modest changes in Ca^2+^ levels used should not have markedly affected excitability. The network effects of low and high Ca^2+^ Ringer also persisted in corrected Ringer to avoid surface screening and when KCa channels were blocked by apamin, arguing against these effects underlying the network changes. However, there was a case of a difference in the effects of corrected and uncorrected low Ca^2+^ Ringer (see Figures 4E,F), which leaves open the potential for non-synaptic effects. The corrected Ringer changes will alter Mg^2+^ levels, and thus while this could avoid surface screening, it now adds the complication of potential changes in NMDA conductance (Mayer et al., 1984). That this was probably not the case was suggested by the absence of a significant effect on the EPSP half-width and the similar network effects of corrected and uncorrected Ringer. The lack of a significant effect of changes in RMP, excitability, and NMDA conductance either individually or in combination, was supported by the lack of effect on cord stimulation-evoked ventral root activity (the exception was uncorrected low Ca^2+^ Ringer, where cord excitability was increased, possibly as a result of reduced surface screening).

Brainstem-evoked locomotor activity would avoid the issues surrounding NMDA-evoked activity, but the variability of the activity evoked by episodic electrical stimuli would make it difficult to determine the effects of the Ringer changes. Tonic pharmacological stimulation of the brainstem could drive fictive activity through descending excitatory inputs, but the issue here is that the input would also be affected by the Ringer changes. As fictive or actual activity is dependent on the strength of the descending drive changes in the reticulospinal synaptic input would itself change the network output, again making it difficult to ascribe effects to changes at the network level. NMDA-evoked activity has the advantage that if left for a long enough time to become stable, as documented in the Methods, it provides a constant rhythmic pattern on which the network effects of Ringer manipulations can be tested.

A further problem with changing Ringer Ca^2+^ is that it will affect all synapses. This is an issue for the proposed hemisegmental depressing excitatory and facilitating feedback inhibitory circuits. While these could act synergistically as burst terminating factors, they will be differently affected by the Ringer changes (e.g., high Ca^2+^ will increase depression of the excitatory group and reduce facilitation of the inhibitory feedback circuit). To overcome this the network was peeled back to its minimum (a hemisegment in strychnine and apamin; see Senn et al., 1996), which as far as we know leaves only connections between EINs and EIN inputs to motor neurons. A caveat here is that EIN connections undergo functional changes after lesioning that become statistically significant 30 min after hemisectioning (Hoffman and Parker, 2010), and thus these changes would be present when the experiments were performed (>2 h after hemisction).

The changes in Ringer Ca^2+^ also altered the amplitude of the first PSP in the spike train. In high Ca^2+^ there was a non-significant increase and in low Ca^2+^ Ringer a significant reduction of the EPSP amplitude. Ideally activity-dependent plasticity should be changed without affecting the initial PSP amplitude, as can be done in simulations, to avoid changes in initial synaptic drive and of summed synaptic inputs over spike trains (see Figure 2E). High Ca^2+^ Ringer increased depression from a non-significantly larger initial PSP, and low Ca^2+^ evoked facilitation from a significantly smaller initial PSP. The development of depression and facilitation meant that by the end of the spike train the summed synaptic input matched control levels, but over the early part of the spike train there were differences in the summed synaptic input. If the frequency of fictive locomotion was directly proportional to glutamate concentration (see Brodin et al., 1985), changes in summed inputs as a result of initial EPSP effects could alter the burst frequency independently of activity-dependent synaptic plasticity. However, a wide range of fictive locomotor frequencies are evoked by a single glutamate agonist concentration (see Parker et al., 1998; this can also be seen by comparing data across or within studies, e.g., Tégner et al., 1993; Figures 1, 2), arguing against a simple relationship between glutamatergic levels and burst frequency. High and low Ca^2+^ Ringer will also affect inhibitory synapses arguing against a simple increase/decrease in excitatory drive. The general lack of changes in cord-evoked ventral root activity also suggests against significant changes in network excitability caused by changes in the initial PSP amplitude, suggesting that the network changes reflect activity-dependent synaptic properties.

While the use of altered Ringer Ca^2+^ to address the network relevance of short-term plasticity invites these, and other, caveats and criticisms, it seems better to consider the potential issues rather than negate the use of the only approach open to investigate a potentially important network aspect. In other spinal networks open to genetic or optogenetic approaches there may eventually be opportunities to test these synaptic effects more precisely. The simplest interpretation of the experimental effects shown here is that high Ca^2+^ enhanced and low Ca^2+^ reduced the depression of the hemisegmental EIN group, and that the depression of the glutamatergic drive can act as a burst terminating factor (see also Senn et al., 1996; Fedirchuk et al., 1999; Tabak et al., 2001).

The network model supported this role for the depression of excitatory inputs. The feedback inhibitory circuit wasn't needed for bursting, in the model or experimentally, but in both cases its removal increased the burst frequency. While the inhibitory feedback circuit could in principle provide a parallel burst termination mechanism, the experimental analysis in the hemicord in strychnine suggested it was of less importance than depression of the EIN group. However, the model suggested that feedback inhibition markedly widened the active range for Depression and D_Rec_, which thus allowed bursting from levels that it could not occur with depression alone. Feedback inhibition may thus regulate the influence of excitatory synaptic depression. Support for this was not sought in the current experimental analysis, but this could be tested by using larger changes in Ringer Ca^2+^ to increase depression of the EIN group to see if the network tolerance for depression was reduced in strychnine. The simulation further suggests this should occur especially at high frequencies.

There are various aspects that could be addressed in detailed network models. Data exists on various aspects of activity-dependent synaptic effects in lamprey network synapses (Parker and Grillner, 1999, 2000; Parker, 2000, 2003a,b; see Silberberg et al., 2005; Parker, 2007 for summaries). However, apart from the results of the first of these analyses (Parker and Grillner, 1999) in a lamprey network simulation (Kozlov et al., 2001), activity-dependent synaptic plasticity has not been included as a model parameter in lamprey simulations. The lack of reference to these effects since the Kozlov et al. (2001) simulation argues against the claim that modeled synaptic properties “very closely resemble those of their biological counterparts” (Kozlov et al., 2009). It would be useful to test these effects in larger network simulations to allow populations of cells to be examined that could allow the introduction of variability of the synaptic effects to be examined. In addition to the role of the inhibitory motif outlined above, the major aspect to be addressed experimentally in systems where activity-dependent synaptic effects are studied is how effects translate from quiescent to active networks. Assumptions that effects are the same may not be justified (e.g., network activity influences activity-dependent synaptic plasticity; Parker, 1995, 2000; Wang and Kaczmarek, 1998; see Zucker and Regehr, 2002). Addressing this will require finding ways of examining synaptic properties during ongoing network activity, something that is currently difficult even in simpler systems (see Nadim et al., 1999).

## Author contributions

YJ performed simulations and experiments and contributed to the writing of the paper. DP performed experiments and wrote the paper.

### Conflict of interest statement

The authors declare that the research was conducted in the absence of any commercial or financial relationships that could be construed as a potential conflict of interest. The reviewer Michael Hendricks and handling Editor Edward S. Ruthazer declared their shared affiliation, and the handling Editor states that the process nevertheless met the standards of a fair and objective review.
